# *BdorOBP2* plays an indispensable role in the perception of methyl eugenol by mature males of *Bactrocera dorsalis* (Hendel)

**DOI:** 10.1038/s41598-017-15893-6

**Published:** 2017-11-21

**Authors:** Huan Liu, Xiao-Feng Zhao, Lang Fu, Yi-Ye Han, Jin Chen, Yong-Yue Lu

**Affiliations:** 1 Department of Entomology, South China Agricultural University, Guangzhou, 510642 China; 2 College of Plant Protection, Fujian Agriculture and Forestry University, Fujian, 350002 China

## Abstract

*Bactrocera dorsalis* (Hendel) is a fruit-eating pest that causes substantial economic damage to the fresh produce industry in tropical and sub-tropical countries. Methyl eugenol (ME) is a powerful attractant for mature males of *B. dorsalis*, and has been widely used for detecting, luring and eradicating *B. dorsalis* populations worldwide. However, the molecular mechanism underlying the olfactory perception of ME remains largely unknown. Here, we analyzed the differential proteomics profiling of the antennae between ME-responsive and ME-non-responsive males by using isobaric tags for relative and absolute quantitation (iTRAQ). In total, 4622 proteins were identified, of which 277 proteins were significant differentially expressed, with 192 up-regulated and 85 down-regulated in responsive male antennae. Quantitative real-time PCR (qRT-PCR) analysis confirmed the authenticity and accuracy of the proteomic analysis. Based on the iTRAQ and qRT-PCR results, we found that the odorant-binding protein 2 (*BdorOBP2*) was abundantly expressed in responsive male antennae. Moreover, *BdorOBP2* was significantly up-regulated by ME in male antennae. Mature males showed significantly greater taxis toward ME than did mature females. Silencing *BdorOBP2* reduced mature males’ responsiveness to ME. These results indicate that *Bdor*OBP2 may play an essential role in the molecular mechanism underlying *B. dorsalis* olfactory perception of ME.

## Introduction

The Oriental fruit fly, *Bactrocera dorsalis* (Hendel), is one of the most destructive fruit/vegetable-eating agricultural pests in the world, particularly in Asiatic countries such as China^1,2^. *B. dorsalis* is a typical polyphagous pest with larval stage feeding, multiple mating, long life span and great fecundity of adults, capable of causing severe damage to more than 250 commercially-valuable tropical and subtropical crops, especially some staple fruits, including mango, guava, orange, carambola, jujube, loquat, peach, etc.^3–5^. Considering the mining habits of the larval stage, pest control strategies focus mainly on the adults. Trapping technology based on flavor cues is an important strategy for controlling these fruit flies. The natural phenylpropanoid, methyl eugenol (ME), has been used widely and effectively for monitoring and eradicating males of *B. dorsalis* populations worldwide^6–8^. ME functions as a precursor for the synthesis of a sex pheromone in *B. dorsalis* males, which makes those males more attractive to females^9^. However, there are human health implications. ME is considered carcinogenic to humans by the National Toxicology Program (NTP), US Department of Health and Human Services, making it not suitable for long-term use^10–14^. In addition, ME can trap sexually mature male flies, but it fails to attract immature males or females. Although, several analogues of ME have been developed^13,15–17^, the trade-off with male attractancy makes them an unlikely improvement over ME for detecting the oriental fruit fly in the field. Therefore, clarifying the molecular mechanism underlying the olfactory perception of ME should provide much-needed information for the developing of efficient, simple, green and sustainable lures for monitoring and controlling *B. dorsalis* pest populations.

Insects have mastered the art of using semiochemicals, encompassing pheromones, kairomones, repellents and attractants, as communication signals and to process stimuli from the environment, including the whereabouts of food sources, reproductive partners, oviposition sites, hosts, or for detecting predators from a distance^1,2,18^. Insect olfaction is highly sensitive so as to reliably discriminate among semiochemicals to mediate these important behaviors. Not surprisingly, the olfactory system of insects is sophisticated and involves many kinds of proteins: namely, odorant-binding proteins (OBPs), chemosensory proteins (CSPs), odorant receptors (ORs), ionotropic receptors (IRs), odorant-degrading enzymes (ODEs) and sensory neuron membrane proteins (SNMPs)^19,20^. The initial steps in odor detection involve the binding of an odor to the ORs displayed on the dendrites of olfactory sensory neurons (OSNs)^21,22^. Insect OBPs that are located in the aqueous sensillum lymph of the antennae are thought to be the first proteins binding the odors to facilitate the transport of hydrophobic odorants through the aqueous surroundings to peripheral ORs, thereby starting the signal transduction cascade leading to behavioral outputs^23–27^. Recently, the use of OBPs as a molecular target for rapidly elucidating behaviorally active compounds and screening of novel species-specific repellents or attractants in insects is gaining attention^28–30^. It has been demonstrated that OBP1 plays an essential role in mediating indole recognition in the antennae of female *Anopheles gambiae* ^31^. Additionally, knock-down of OBP1 in the mosquito *Culex quinquefasciatus* reduced its antennal response to several oviposition attractants^32^. By using a molecular docking and molecular dynamics simulation, Kempraj *et al*.^18^ studied 25 semiochemicals’ binding potential to a GOBP of *B. dorsalis* for predicting and screening behaviorally active compounds. Silencing *Orco* and *OBP83a-2* decreased the ability of *B. dorsalis* adults to find semiochemical lures in a timely manner^14,33^. In this regard, this unique research strategy may help chemical ecologists in their prospecting of active semiochemicals for eco-friendly use in pest management practices.

Isobaric tags for relative and absolute quantitation (iTRAQ), a new technique that has become popular in proteomic studies in recent years, can provide extremely reliable quantitative comparisons and identifications of molecular candidates among complex biological samples^34,35^. To date, this approach has been successfully used in many organisms, such as *Bemisia tabaci* ^36^, *Apostichopus japonicas* ^35^, *Locusta migratoria* ^37^, and *Culex pipiens pallens* ^38^, to name a few. Interestingly, experiments have revealed that the proportion of males with non-taxis to ME could be increased persistently via several generations of artificial selection under laboratory conditions^11,39^. These results suggest that the characteristics of defective olfactory sensory physiology to ME of non-responsive individuals may be passed on to offspring. Therefore, we speculated that the responsiveness to ME of male *B. dorsalis* flies is controlled by those genes associated with olfactory processing. In the present study, we utilized the iTRAQ approach combined with LC-MS/MS analyses to investigate the differential proteomics profiling of the antennae between responsive and non-responsive *B. dorsalis* mature males to ME. We identified an odorant-binding protein 2 (OBP2) highly expressed in the antennae of responsive male flies and determined its molecular characterization, expression profile, and function in ME perception.

## Results

### Non-responders to ME in the select and control lines

**Figure 1:**
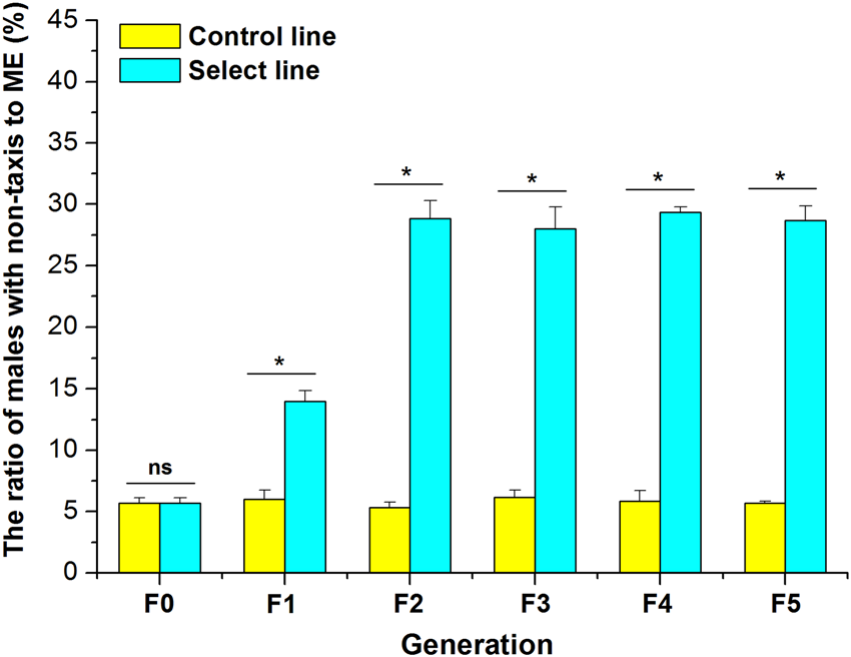
Proportion of *Bactrocera dorsalis* male flies that were non-responsive to ME for each generation. The presence of * above a column pair marks a significant difference (*t-test*, *P* < 0.05). All the experiments were performed in triplicate. Bars represent mean ± SE values.

The frequency of non-responsive males to ME in the select and control lines for each generation are illustrated in Fig. 1. In the select lines, the mean proportions of non-responders in the first generation (F1) and second generation (F2) were respectively 14.0 ± 0.9% and 28.8 ± 1.5%, both significantly higher than those in the control lines, which were 6.0 ± 0.8% (*t* = 13.86; *df* = 2; *P* = 0.0052) and 5.3 ± 0.4% (*t* = 14.62; *df* = 2; *P* = 0.0046), respectively. These results indicated that the responsiveness of males to ME evidently decreased via the selection experiments. Further, the proportion of non-responders increased rapidly and remained consistently high (28.0% – 29.3%) for the select males between the second and fifth generations. By contrast, the proportion of non-responders in the control line were not statistically different among all generations (*F* = 0.24; *df* = 5; *P* = 0.9375).

### Identification of differentially expressed antennal proteins between the responsive and non-responsive male flies

**Figure 2:**
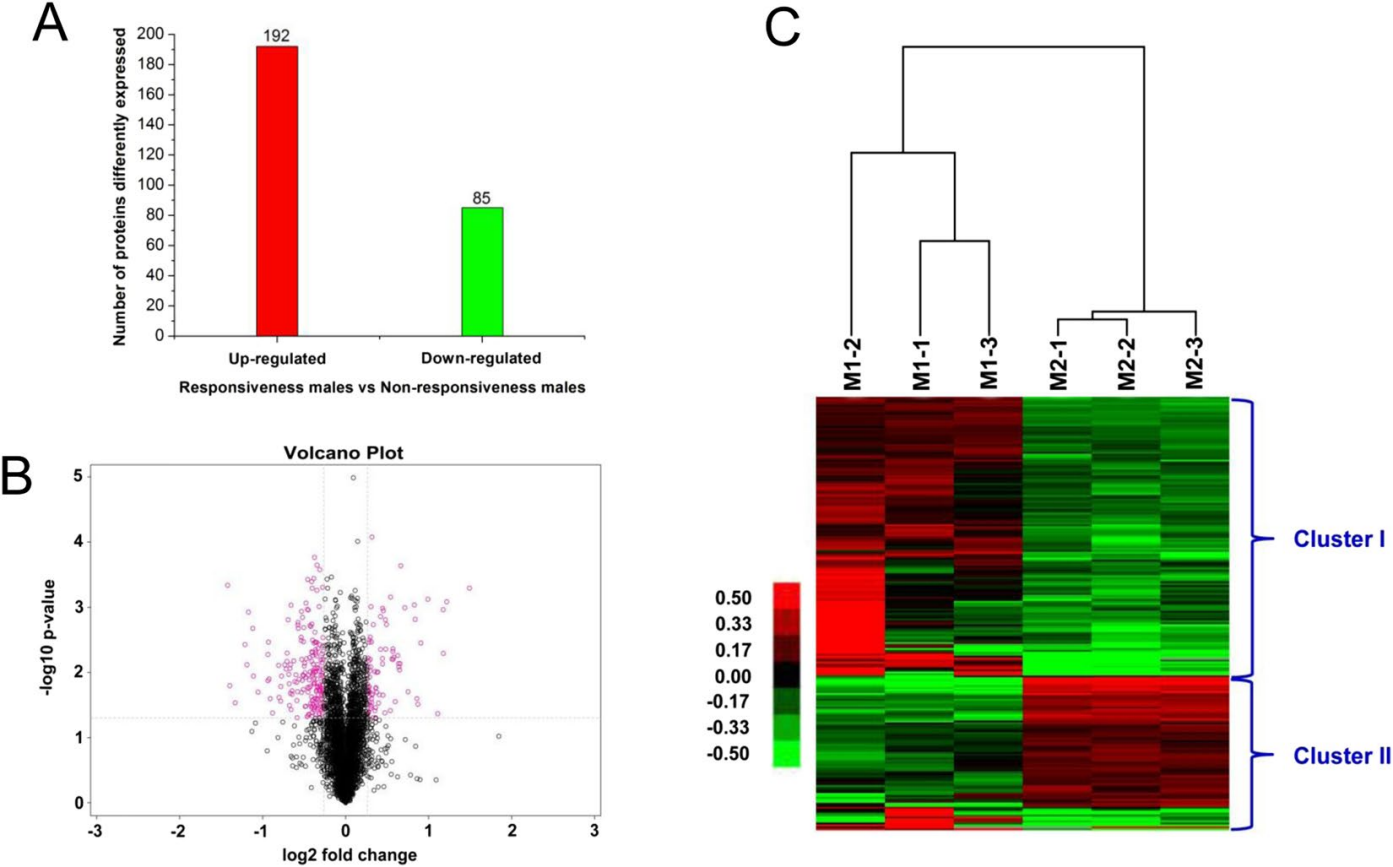
Statistical analysis of the differential expression of proteins in the responsive and non-responsive males’ antennae of *Bactrocera dorsalis* files. (**A**) The classification of differential abundance of proteins. (**B**) The change expression level of global proteins in the responsive and non-responsive male antennae (only the proteins with log2 fold changes ≥1.20 or ≤0.83 were colored red in the volcano plot). (**C**) A heat map analysis of differential protein expression profiles from three replicates (M1 and M2 represent the responsive and non-responsive males, respectively). Cluster I red indicates those proteins that were significantly up-regulated in the responsive males. Cluster II green indicates significantly down-regulated proteins in the responsive males.

The total mass spectra number detected in the male antennae proteomes was 266460, representing 48059 peptide spectra and 26679 distinct peptides. The results also indicated that a total of 4622 proteins were successfully identified (Table S1). The global expression changes of all proteins with quantitative iTRAQ ratios are shown in Fig. 2B; the red circles are proteins that exhibited a differential expression pattern in responsive males compared with the non-responsive males. A heat map (Fig. 2C) was constructed according to the data of 277 differential proteins between responder and non-responder. Compared with the non-responsive males, 192 proteins showed an increased abundance, and 85 proteins showed a decreased abundance in the responsive males antennae (with a fold change of ≥1.20 or ≤ 0.83, *P* value < 0.05; FDR < 0.01) (Fig. 2A). Among the differentially expressed proteins, we identified five proteins associated with insect olfactory recognition, including four odorant binding proteins, and one odorant receptor (Table 1). The respective mass spectra and peptide sequences of these five olfactory proteins are shown in Figure S1. Notably, *BdorOBP2* was up-regulated by 1.34-fold in the responsive males’ relative to the non-responsive males’ antennae. Further, proteins related to energy regulation, protein transportation and binding were also differentially expressed between the responders and non-responders (Supplementary Dataset S1).

**Table 1:** Identification of proteins that are differentially displayed in non-responsive and responsive *B. dorsalis* male antennae (Partial).

Accession Number	Protein Description	Coverage (%)	No. of unique peptides	No. of peptides	Theor. pI/MW (kDa)	Non-responsiveness/Responsiveness	Student’s *t*-test *P* value
**Purine metabolism**							
A0A034WES2	Head-specific guanylate cyclase	1.48	1	1	6.075/75.578	0.691	0.00116
A0A034WMW0	DNA polymerase delta small subunit	2.34	1	1	6.252/48.460	0.690	0.00323
A0A034W568	Xanthine dehydrogenase	2.20	1	1	7.210/91.164	0.608	0.00882
A0A034VHC1	cAMP-specific 3′,5′-cyclic phosphodiesterase	5.68	2	2	5.224/70.722	0.622	0.0107
A0A034W7G2	DNA primase large subunit	1.68	1	1	7.723/62.851	0.669	0.0197
A0A034W8Q9	Trifunctional nucleotide phosphoesterase protein YfkN	5.61	2	2	6.075/65.887	0.667	0.0495
**Olfactory transporter**							
A0A0G3Z7T5	Odorant binding protein 44a	62.24	12	12	8.207/16.514	1.361	0.00103
A0A034WGF4	Putative odorant-binding protein A5	53.33	5	5	8.397/13.751	1.260	0.00184
A0A0G2UEY4	Odorant receptor 94b	3.03	1	1	8.558/46.003	1.683	0.0198
A0A0G2UEV0	Odorant binding protein 69a	48.3	1	7	5.275/16.531	1.451	0.0385
S5R7H8	Odorant binding protein 2	36.23	4	5	5.389/14.930	0.744	0.00980
A0A034VRC9	Calcium/calmodulin-dependent 3′,5′-cyclic nucleotide phosphodiesterase 1 C	9.16	3	3	5.592/62.712	0.822	0.0189
**Basal transcription factors**							
A0A034VQF4	General transcription factor IIH subunit 4	2.65	1	1	8.778/55.785	1.200	0.0152
A0A034VQ28	Transcription initiation factor TFIID subunit 5	1.02	1	1	6.392/76.879	1.208	0.0186
A0A034V527	Cyclin-dependent kinase 7	7.06	1	1	8.353/38.518	0.752	0.0237
A0A034WL27	Transcription initiation factor TFIID subunit 9	4.14	1	1	9.159/28.839	0.783	0.0306
A0A034W993	Transcription initiation factor TFIID subunit 6	3.4	2	2	7.254/67.867	0.734	0.0446
**Endocytosis**							
A0A034WV96	Actin-related protein 2/3 complex subunit 3	11.86	2	2	8.631/20.485	0.718	0.000861
A0A034V1T6	WASH complex subunit FAM21-like protein	0.83	1	1	4.856/182.451	0.762	0.0105
**Ribosome biogenesis in eukaryotes**							
A0A034WFT2	Casein kinase II subunit beta	10.36	1	1	5.440/25.671	0.613	0.00678
A0A034VPR3	Elongation factor Tu GTP-binding domain-containing protein 1	1.05	1	1	6.100/116.739	0.522	0.0134
A0A034VB78	WD repeat-containing protein 75	1.52	1	1	7.386/98.023	0.782	0.0195
A0A034V243	HEAT repeat-containing protein 1-like protein	0.8	1	1	6.991/169.835	0.666	0.0269
**MAPK signaling pathway**							
A0A034V9V5	Heat shock protein 70	14.19	4	9	5.656/68.597	1.244	0.0289
A0A034W8P6	Protein E(Sev)2B	13.74	3	3	5.491/24.446	0.771	0.000172
A0A034VM24	Ras GTPase-activating protein 1	4.25	2	2	7.196/104.043	0.762	0.00984
**Phosphatidylinositol signaling system**							
A0A034VKU6	Myotubularin-related protein 3	0.55	1	1	5.427/139.846	0.776	0.00987
A0A034VK69	Phosphatidylinositol 3,4,5-trisphosphate 3-phosphatase and dual-specificity protein phosphatase PTEN	1.12	1	1	7.708/98.570	0.774	0.0328
**PI3K-Akt signaling pathway**							
A0A034WIV7	Phosphoenolpyruvate carboxykinase (GTP)	2.53	1	1	6.443/39.978	0.527	0.0126
**Insulin signaling pathway**							
A0A034V7R7	Guanine nucleotide-releasing factor 2	0.81	1	1	6.815/150.361	0.532	0.0252
**PPAR signaling pathway**							
A0A034WB65	Putative medium-chain specific acyl-CoA dehydrogenase, mitochondrial	59.22	1	6	8.119/11.576	0.721	0.00616
A0A034WWV5	Putative glycerol kinase 3	2.35	1	1	5.872/66.201	0.737	0.0173
**RNA transport**							
A0A034WNU0	Nuclear cap-binding protein subunit 2	7.1	1	1	8.148/17.893	1.204	0.0433
A0A034VSB0	Translation initiation factor eIF-2B subunit epsilon	6.03	2	2	7.035/39.087	0.826	0.0155
A0A034W1I4	Exportin-5	5.35	2	2	6.742/95.272	0.817	0.0217
**Spliceosome**							
A0A034VJC6	ATP-dependent RNA helicase DDX42	3.82	2	2	6.786/86.863	0.802	0.000977
**Peroxisome**							
A0A034WF74	Putative fatty acyl-CoA reductase CG5065	6.27	1	1	8.690/60.453	0.634	0.000929
A0A034WGR2	PXMP2/4 family protein 4	3.08	1	1	8.807/26.688	0.673	0.00210
A0A034WEU0	Superoxide dismutase (Cu-Zn)	5.47	1	1	7.005/29.844	0.823	0.0191

Notes: Accession number is the unique number given to mark the entry of a protein in the database UniProt. Protein description is given when proteins were identified by MALDI-TOF/MS. The taxonomy is *Bactrocera dorsalis*. Coverage is the ratio of the number of amino acids in every peptide that matches with the mass spectrum divided by the total number of amino acids in the protein sequence. Theoretical molecular weight (MW) and isoelectric point (pI) of the identified proteins were retrieved from the protein database of NCBInr. *P*-value ≤ 0.05 is considered as differentially expressed proteins.

### GO annotation of the differentially expressed proteins

**Figure 3:**
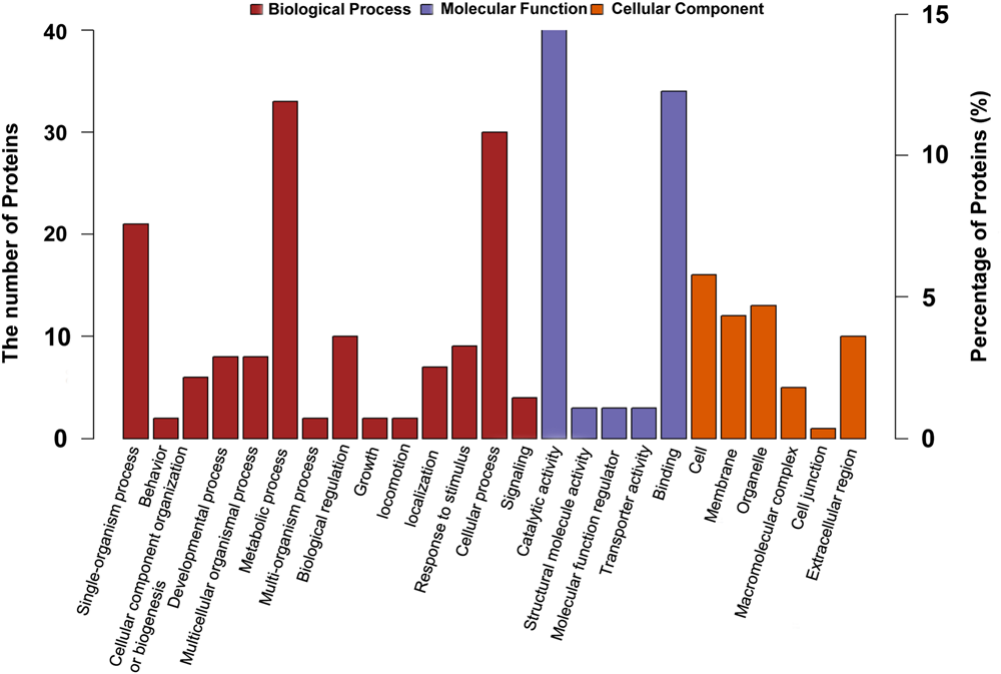
Gene Ontology classifications of antennal differentially expressed proteins as identified by iTRAQ in the responsive and non-responsive *Bactrocera dorsalis* male files. The *y*-axis (left) represents the protein number, and the *y*-axis (right) represents the percentages of the proteins identified. The functional assignments to biological processes, molecular functions, and cellular components are shown from the number of proteins and corresponding proportion converted.

For insights into the functional categories that were altered between the responsive and non-responsive male antennae, the Gene Ontology (GO) database and UniProt knowledge base were used to categorize the identified differential proteins. They covered a wide range of biological processes, molecular functions and cellular components, which could be classified into 14, 5, and 6 subcategory groups, respectively (Fig. 3). Specifically, the largest group within the biological process category was that of metabolic processes, followed by cellular processes and single-organism processes, whereas catalytic activity and binding were the most common categories for molecular function. The cellular component functions of these proteins were mainly related to the cell, organelles, membrane and extracellular region. Upon further investigation by the GO Enrichment analysis (*P* value < 0.05), all of the following were significantly affected: aminoglycan metabolic process, cuticle pigmentation, receptor binding, small molecule binding, neuropeptide hormone activity, protein heterodimerization activity, hydrolase activity acting on glycosyl bonds, growth factor receptor binding, and extracellular region (Figure S2).

### KEGG pathway analysis

To identify the active biological pathways in the ME perception process, we mapped the detected proteins to reference canonical pathways in the Kyoto Encyclopedia of Genes and Genomes (KEGG) by using KAAS (KEGG Automatic Annotation Server). We obtained 174 maps using the proteins, and the predictions for the most differentially expressed proteins suggest that they are involved in 19 pathways (Figure S3). The pathways with the greatest representation by unique proteins were those of purine metabolism, olfactory transporter and basal transcription factors, with six, six and five proteins respectively. Compared with non-responsive males, six proteins with either a greater (*BdorOBP2*, nucleotide phosphodiesterase) or lower (*BdorOBP44a*, *BdorOBP69a*, *BdorOBA5*, *BdorOR94b*) abundance in the responsive males’ antennae are related to olfactory transport (Table 1). The annotations provide a valuable resource for investigating the specific processes, functions and pathways involved in the ME perception process.

### Validation of differentially expressed proteins by qRT-PCR

**Figure 4:**
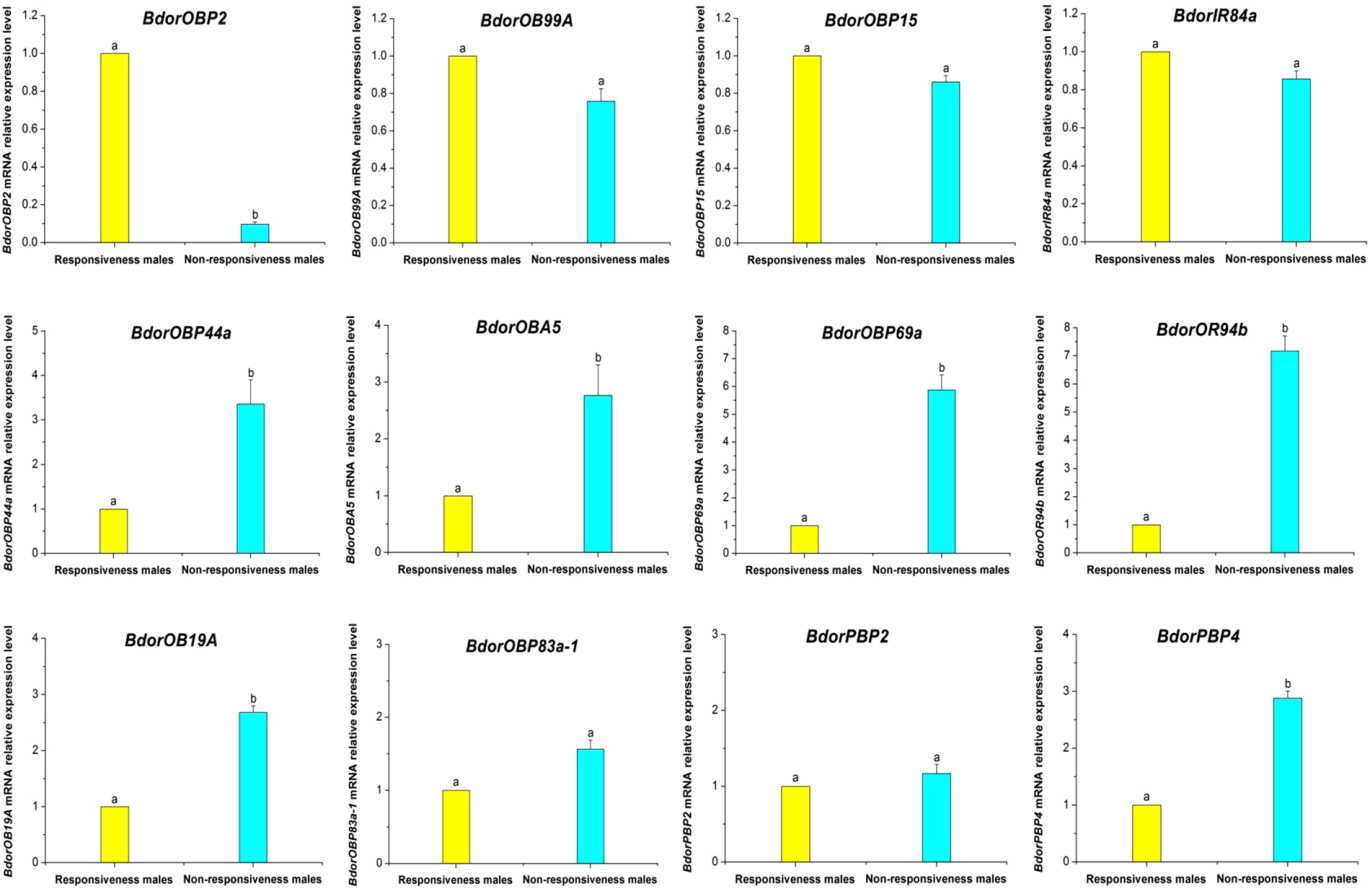
Quantitative real-time PCR to analyse the candidate olfactory proteins transcript levels in antennae of responsive and non-responsive *Bactrocera dorsalis* male files. Different letters between the columns indicate a significant difference in the expression level of olfactory genes (*t-test*, *P* < 0.05). Three biological replicates were performed. Bars represent mean ± SE values.

To validate the results from the proteomic analysis, genes encoding differentially expressed levels of olfactory proteins in the antennae between the responsive and non-responsive males were detected by qRT-PCR (Fig. 4). Results showed that the expression of *BdorOBP2* in the antennae of responsive males was remarkably higher (*t* = 97.87; *df* = 2; *P* = 0.0001). The transcript levels of *BdorOBP44a*, *BdorOBA5*, *BdorOBP69a*, *BdorOR94b*, *BdorOB19A and BdorPBP4* were respectively 3.36, 2.76, 5.87, 7.17, 2.68 and 2.88 times higher in the non-responsive than in responsive male antennae. For the other genes tested, *BdorOB99A*, *BdorOBP15*, *BdorOBP83a-1*, *BdorPBP2* and *BdorIR84a* were expressed at similar levels in the antennae of the non-responsive and responsive males. The mRNA results by qRT-PCR showed that 10 genes: *BdorOBP2*, *BdorOBP44a*, *BdorOBA5*, *BdorOBP69a*, *BdorOR94b*, *BdorOB99A*, *BdorOBP15*, *BdorOBP83a-1*, *BdorPBP2* and *BdorIR84a* were consistent with the protein expression analyzed by iTRAQ. However, two proteins, *BdorOB19A* and *BdorPBP4* expressed no significant difference at the proteomic level, which pointed to mRNA-protein expression inconsistencies.

### Phylogenetic analysis of *BdorOBP2*

**Figure 5:**
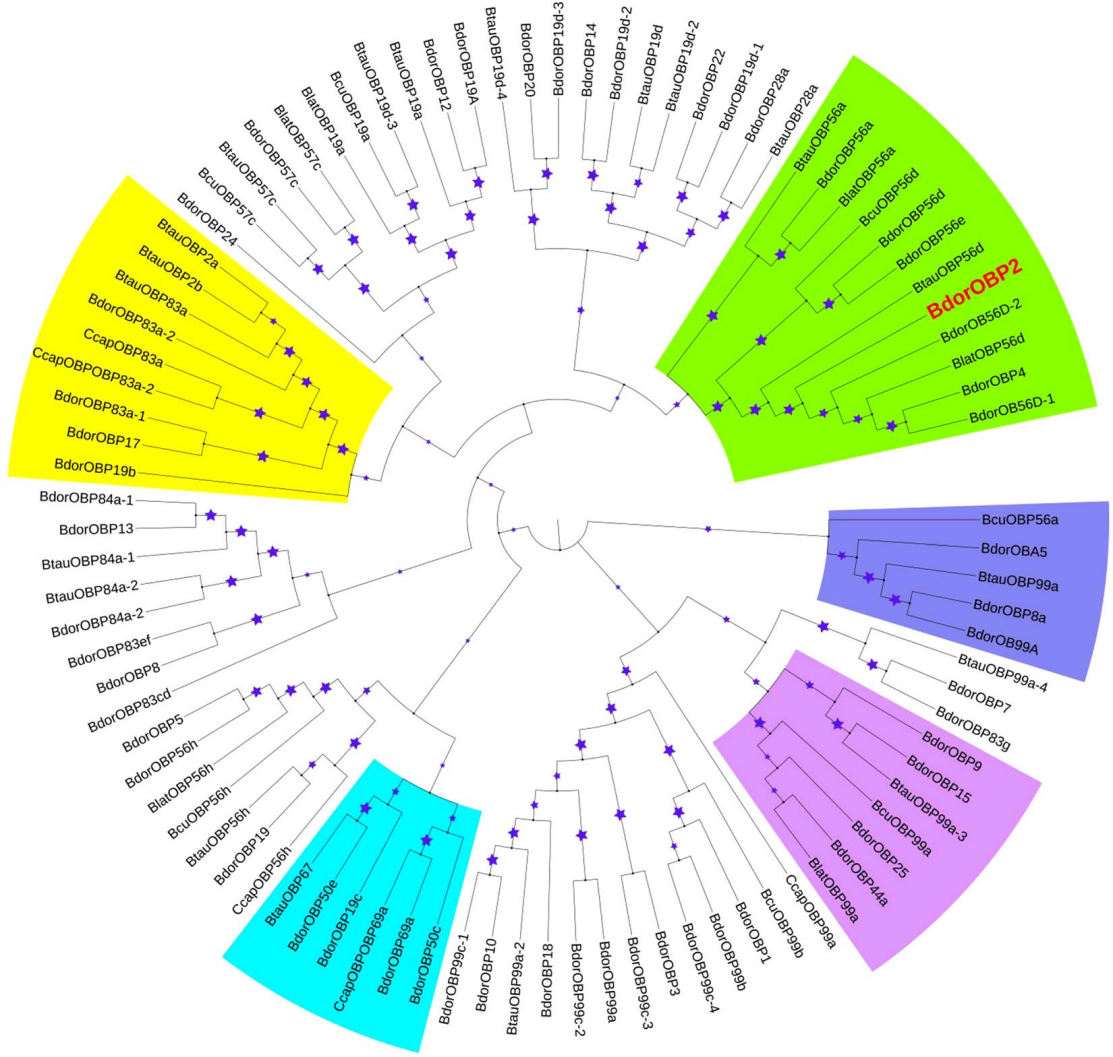
Phylogenetic relationships of *Bactrocera dorsalis*, *Bactrocera tau*, *Bactrocera cucurbitae*, *Bactrocera latifrons* and *Ceratitis capitata* OBP proteins. Bootstrap values greater than 50% (1000 replications) are displayed.

The full-length *BdorOBP2* complementary DNA (cDNA) consisted of 621 nucleotides (nt), with an open reading frame (ORF) of 417 bp, and an encoded polypeptide of 139 amino acids. To determine the phylogenetic relationship between *BdorOBP2* and the 91 OBPs reported in *Bactrocera tau*, *Bactrocera cucurbitae*, *Bactrocera latifrons* and *Ceratitis capitata*, a neighbor-joining tree was constructed (Fig. 5). As expected, the *BdorOBPs* clustered together with orthologous OBPs from the Tephritidae species with the best BLASTP hit. The classic OBPs from *B. dorsalis* shared phylogenetic relationships with the OBP homologs from the Tephritidae species. Notably, *BdorOBP2* is clustered in a branch with the orthologous gene *OBP56*.

### Sex and age affect the taxis of flies to ME and *BdorOBP2* expression

**Figure 6:**
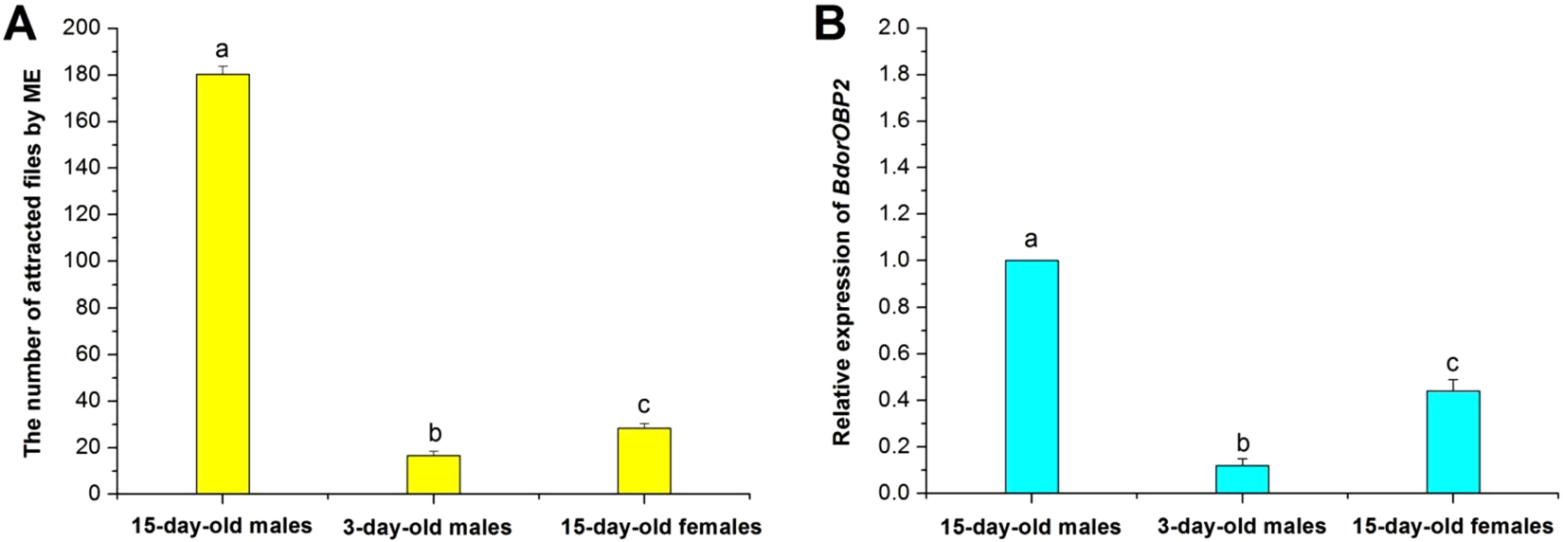
The attractive effect of ME to *Bactrocera dorsalis* flies and the expression level of *BdorOBP2* in adult antennae. (**A**) The attractive effect of ME to immature and mature flies. (**B**) The expression level of *BdorOBP2* in the antennae of immature and mature flies. Different letters among the columns indicate significant differences by ANOVA (*P* < 0.05). All the experiments were performed in triplicate. Bars represent mean ± SE values.

Adult sex and age had a clear influence on the flies’ taxis to ME. As Fig. 6A shows, responsiveness increased with age, with newly emerged adults presenting the lowest taxis. The 15-day-old male responders numbered 180.3 ± 3.3, which was significantly higher than in 3-day-old males (16.7 ± 1.8) and 15-day-old females (28.3 ± 2.0) (*F* = *951.89; df* = *2*; *P* < *0.0001*). Furthermore, in contrast to the low expression observed in the 3-day-old male flies, *BdorOBP2* was highly expressed in 15-day-old males, being 8.33-fold greater than for 3-day-old males and 2.27-fold that of the 15-day-old females (*F* = 117.31; *df* = 2; *P* = 0.0003) (Fig. 6B).

### Confirmation of ME exposure for up-regulated *BdorOBP2* expression

**Figure 7:**
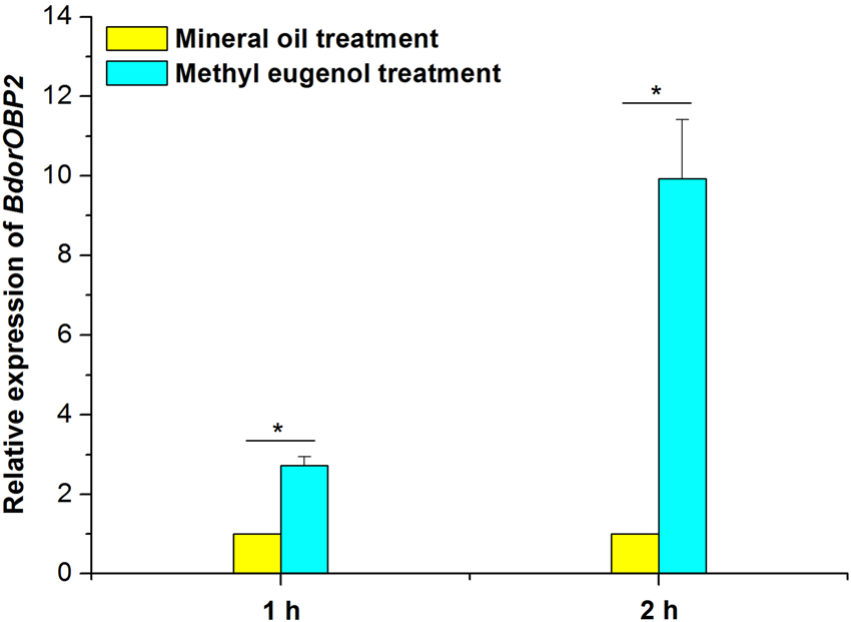
ME regulates on the expression of *BdorOBP2* in the antennae of *Bactrocera dorsalis* males. The presence of * above a column pair denotes a significant difference (*t-test*, *P* < 0.05). All the experiments were performed in triplicate. Bars represent mean ± SE values.

The ME treatment had a clear, positive influence on *BdorOBP2* expression. As Fig. 7 shows, this was up-regulated in the mature male antennae to a level 2.72-fold greater than that under the mineral oil treatment after 1 h (*t* = 7.80; *df* = 2; *P* = 0.0161), a difference that reached c. 9.92-fold after 2 h (*t* = 5.93; *df* = 2; *P* = 0.0273).

### *BdorOBP2* mediated the taxis of male flies to ME

**Figure 8:**
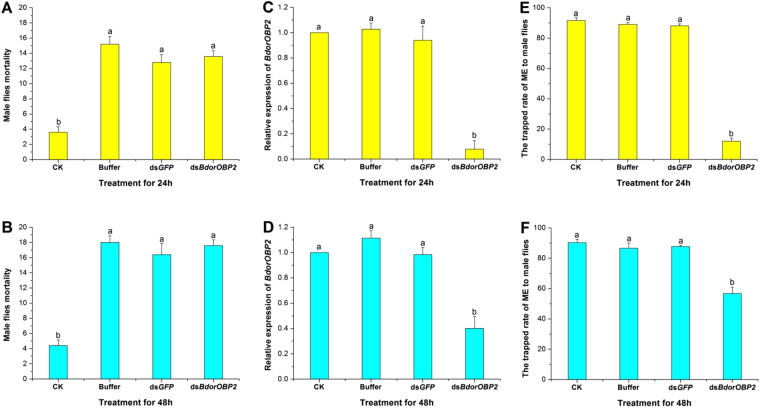
Effects of RNA interference on the *Bactrocera dorsalis* male flies’ mortality, *BdorOBP2* expression in antennae and responsiveness to ME. (**A**,**B**) Mortality rate of male flies in the ds*BdorOBP2* treatment in the 24 h and 48 h bioassays was calculated. Normal control males were injected with the same amount of ds*GFP* and Buffer. Blank control groups (CK) were normally reared. (**C**,**D**) The qRT-PCR analysis of *BdorOBP2* expression after *BdorOBP2* was silenced by RNAi. (**E**,**F**) The attractive effect of ME to males after *BdorOBP2* was silenced by RNAi. Different letters among the columns indicate significant differences (ANOVA, *P* < 0.05). Five biological replicates were performed. Bars represent mean ± SE values.

The bioassay done on *B. dorsalis* showed that the average mortalities of ds*BdorOBP2*, ds*GFP* and the buffer treatment groups at 24 h were 13.6 ± 0.8%, 12.8 ± 1.0% and 15.2 ± 1.0% respectively (Fig. 8A) and the mortality increased to 17.6 ± 0.8%, 16.4 ± 1.5% and 18.0 ± 0.9% after 48 h continued treatment (Fig. 8B). However, the mortalities of the blank controls were comparatively much low, at 3.6 ± 0.8% for 24 h (*F* = 38.34; *df* = 3; *P* < 0.0001) and 4.4 ± 0.8% at 48 h (*F* = 33.91; *df* = 3; *P* < 0.0001). Notably, the mortality of the ds*BdorOBP2*-treated male flies was not significantly different from that of the ds*GFP*- and Buffer-treated male flies. The results indicate that the microinjection had a negative impact on *B. dorsalis* adults’ survival.

The effects of RNAi of the *BdorOBP2* precursor transcript on male responsiveness to ME were analyzed. The qRT-PCR demonstrated that the knockdown efficiencies were significant for the *BdorOBP2* mRNA levels at 24 h and 48 h after the male flies had been injected with ds*BdorOBP2* (Fig. 8C,D). After treatment for 24 h, *BdorOBP2* expression decreased approximately 90% compared with those of the ds*GFP*, Buffer and blank control groups (*F* = 37.85; *df* = 3; *P* < 0.0001). Consequently, the *dsBdorOBP2*-treated males were much less trapped by ME than were the ds*GFP*- and Buffer-treated flies. This difference was strongest at 24 h, when the males trapped rate by ME was 12.0 ± 2.0% in *dsBdorOBP2* treatment group, significantly less than ds*GFP-*treated group (88.1 ± 1.4%), Buffer-treated group (89.2 ± 1.2%) and control group (91.7 ± 1.9%) (*F* = 548.80; *df* = 3; *P* < 0.0001) (Fig. 8E). After treatment for 48 h, though the males trapped in the *dsBdorOBP2* treatment group had noticeably increased (to 56.8 ± 4.1%), it was still lower, at c. two-thirds that of the other treatment groups and the blank control (*F* = 23.49; *df* = 3; *P* < 0.0001) (Fig. 8F). These results demonstrated that the male flies could not be significantly trapped by ME after *BdorOBP2* was silenced.

## Discussion

The Oriental fruit fly is a biologically interesting and economically relevant insect herbivore, whose mature males are effectively trapped by ME. Over the past few decades, numerous studies have been focused on the factors influencing the ME-luring efficiency on males, and the metabolic pathways or metabolites of ME after feeding by *B. dorsalis* adults^7,9,12,40^. However, the molecular mechanisms underlying the olfactory perception of ME remain poorly understood. Through the iTRAQ technique, we identified some differentially expressed olfactory proteins of the antennae between responsive and non-responsive *B. dorsalis* males to ME. Among these olfactory proteins, *BdorOBP2* was abundantly expressed in the responsive mature males’ antennae of *B. dorsalis* and its expression level was remarkably up-regulated by ME. Moreover, silencing *BdorOBP2* dramatically decreased the number of attracted males to ME. Taken together, results obtained here indicate that *BdorOBP2* likely has an essential role to play in mediating the taxis of ME to *B. dorsalis* male adults. Hence, our present findings further enrich and deepen our understanding of the molecular mechanism underpinning ME perception by *B. dorsalis* mature males.

Notably, the responsiveness to ME in *B. dorsalis* males was not uniform throughout the day: it peaked in the morning, declined in the afternoon, and it then dropped markedly at dusk^40^. This daily fluctuation in the male responsiveness to ME suggests an inverse relationship with the daily cycle of their mating behaviour. For this reason, we performed all of our behavioural assays in the morning. We found that the proportion of select males non-responsive to ME increases as the generation increases. However, the highest proportion would stabilize at about 28%. Yet more remarkably, the selection experiment did not result in the complete disappearance of the male flies’ tropism to ME. This agrees with previous findings by Shelly^11^ and Guo *et al*.^39^, whose showed that the responsiveness of male *B. dorsalis* to ME could be reduced via artificial selection (6–12 generations) under laboratory conditions. Nonetheless, the laboratory experiment by Zheng *et al*.^14^ demonstrated that a small proportion of males became attracted more slowly, with about 10% of males not even attracted to ME after 24 h. This may be attributed to the individual differences in epigenetics, such as in defective olfactory sensory physiology.

Although the gene and protein databases of *B. dorsalis* were incomplete, 277 proteins were successfully found as differentially expressed between the responsive and non-responsive males’ antennae by iTRAQ technology. The functions of these differentially expressed proteins are likely involved in olfactory transduction, amino acid metabolism, basal transcription factors, MAPK signaling pathway, PI3K-Akt signaling pathway, endocytosis, phosphatidylinositol signaling system, cGMP-PKG sinaling pathway, calcium signaling pathway and PPAR signaling pathway. The calcium/calmodulin-dependent 3′,5′-cyclic nucleotide phosphodiesterase 1 C, which plays a pivotal role in balancing intracellular Ca^2+^/CaM and cGMP signaling^41^, was found abundantly expressed in the antennae of responsive males. It is preliminarily conjectured that this protein may be involved in how *B. dorsalis* males perceive ME, but this needs further investigation. In addition, four OBPs (*BdorOBP2*, *BdorOBP44a*, *BdorOBP69a*, *BdorOBA5*) and one OR (*BdorOR94b*) had significant differential expression between responders and non-responders. But only *BdorOBP2* was highly expressed in the antennae of responsive males. The consistency in the results with the mRNA expression by qRT-PCR analysis implies there is a real significance of *BdorOBP2* in the ME perception by mature male flies. While a diverse pattern between mRNA and protein expression in two genes (*BdorOB19A* and *BdorPBP4*) may reflect the lack of a direct relationship between mRNA and protein expression, it could also be attributed to post-transitional effects and/or other regulatory mechanisms, such as a lack of synchronization^42–44^.

Immature male flies were obviously not attracted to ME, but the highest attraction was observed in 15-day-old males, a result consistent with Karunaratne and Karunaratne^40^. That *BdorOBP2* was abundantly expressed in the mature male antennae agrees with prior findings by Zheng *et al*.^1^. Accordingly, very few of the 15-day-old females were attracted by ME. As a corollary, the expression level of *BdorOBP2* in the antennae of mature male flies was up-regulated by ME in just 1 h, which strongly suggests that *BdorOBP2* upregulation may be closely related to the ME attraction for male adults.

Intriguingly, prior researches have demonstrated that proteins in the GOBP2 class have a conserved sequence across different species, and that they possess high binding activity to major pheromones in several insect species^45–47^. Previous study on Lepidopteran insects has proved that *MsexGOBP2* could be photoaffinity-labeled by the pheromone analogue, (6E, 11Z)-hexadecadienyl diazoacetate^48^. *CsupGOBP2* has a high specificity for a major pheromone component, 11Z-hexadecenal^49^, and *BmorGOBP2* can bind to the *B. mori* sex pheromone component, (10E,12Z)-hexadecadien-1-ol^46^. The long-chained chemical, *trans*-11- tetradecen-1-yl acetate, a sex pheromone component of *Loxostege sticticalis* Pyralidae, has a high binding affinity to *LstiGOBP2* ^47^. Silencing of *AgOBP2* impaired the host recognition and detection of oviposition attractants in *Aphis gossypii* Glover. Simultaneously, OBP2 could potentially serve as a practicable target for RNAi-mediated gene silencing in the pest control of Hemipteran insect pests^50^. In the present study, silencing *BdorOBP2* via the injection of dsRNA reduced the attractive effects of ME to mature males. In other words, our results demonstrate for the first time that *Bdor*OBP2 participates in mediating the taxis to ME in male *B. dorsalis* flies, which sheds light on the mechanisms underlying the strong attraction of its males to ME. Meanwhile, the microinjected dsRNA decreased the survival of *B. dorsalis* male adults. A plausible explanation for this important phenomenon is that microinjection caused physical damage leading to the death of the tested flies.

Odorant receptor co-receptor (Orco) is an atypical common odorant receptor. It is highly conserved among different insect species, and is co-expressed with a specific odorant receptor in the endomembrane system in olfactory sensory neurons. The Orco/OR complex confers odor-recognition specificity and promotes the functional reconstitution of the odor-evoked signaling transduction pathway in the sensory neurons^51–53^. In this complex, Orco is suggested to be responsible for the stabilization, localization, and for the correct protein folding of OR, thus making it obligatory for the detection of odorants^54^. In addition, Orco also works as a selective ion channel in the response to common odors, such as aldehydes, ketones, esters, and aromatics^54,55^. *BdOrco* was reportedly up-regulated by the sexual attractant ME and played a critical role in mediating the taxis of *B. dorsalis* males to ME^14^. Furthermore, gene silencing via the microinjection of dsRNA recently confirmed that *BdorOBP83a-2* actively participates in the process of ME detection by *B. dorsalis* ^33^. The phylogenetic tree was generated to show how *BdorOBP2* and *BdorOBP83a-2* belong to different groups of orthologous proteins (Fig. 5). Olfaction in insects is extremely complex, consisting of various interactions of many classes of proteins and effectors to reliably translate any external semiochemicals in the environment, so as to ultimately produce a behavioral response in the insect^56^. Considering our findings alongside other research to date, we preliminary posit that ME odor molecules bind to either *BdorOBP2* or *BdorOBP83a-2*, and then are transferred to OSNs, where *BdOrco* coordinates with a specific OR to bind to the ME odor molecules. Only then is the olfactory signal transduction pathway finally activated.

It is the conventional ORs which are activated by odorants that determine the specificity of the response in the olfactory neurons^51,56^. However, no OR protein that underwent significant up-regulation in the responsive males was identified by iTRAQ in our present study. We speculate that because there is no direct stimulation by ME on males, this did not cause the differential expression of OR proteins. Thus, these OR proteins, which are assumed to operate in the olfactory perception of ME in *B. dorsalis*, await further investigation. However, because we lack *B. dorsalis* genomic information, many proteins cannot yet be annotated or their functions and interactions remain poorly understood, especially for those olfactory-related proteins. Nevertheless, with the gradual completion of genome sequencing of *B. dorsalis*, the molecular mechanism and transport pathway underpinning how ME is able to lure mature male flies will become clearer.

## Materials and Methods

### Ethics statement

No specific permits were required for the described field studies, and no specific permissions were required for these locations/activities. We confirm that these locations are not privately owned or protected in any way and that the field studies did not involve endangered or protected species.

### Insect

The *B. dorsalis* genetic sexing strain (GSS) had been maintained in a laboratory for approximately thirty generations in South China Agricultural University. The female pupae are white and the male pupae are brown. The flies were reared at 27 ± 1 °C, 75 ± 1% relative humidity, and a photoperiod cycle of 14 h L/10 h D. Hatched larvae were maintained on an artificial diet includes sugar (8.99%), yeast (15.06%), nipagen (0.15%), sodium benzoate (0.15%), wheat germ oil (0.15%), citric acid (1.70%), and water (73.81%)^57^. Before pupation, larvae were transferred into small plastic boxes with sand. Pupae were kept at 27 ± 1 °C until adults emerged. Adult flies were reared in wooden cages (35 cm by 35 cm by 35 cm) and fed another artificial diet consisting of yeast extract: dry sugar at 1:1 (w/w).

### Artificial selection of non-responsive males to ME

We chose the male flies without taxis to ME according to the methods of Shelly^11^ and Guo *et al*.^39^. The select and control lines were established as follows. At the start of the study, the colony from the *B. dorsalis* genetic sexing strain had been maintained in our laboratory for approximately 30 generations. Male and female new pupae of the same generation were separated and reared in different screen cages (1.0 m × 1.0 m × 1.0 m). When the adult male flies were 15 days old, a fly trap containing 1.0 mL of pure ME (Energy Chemical Company, Shanghai, China) was put into the screen cage. After treatment for 2 h, we removed the trap and discarded the trapped male flies. The remaining male files were kept and reared with unlimited food and water. Three days after the first trapping trial, a second trial was conducted using the same procedure. The rest of male files in the screen cage were considered non-responsive to ME. For the select line, males that failed to get trapped by ME in the double-exposure experiment were used as sires for the next generation; females at the same emergence age were taken randomly from the same generation. For the control line, males and females were chosen haphazardly from among untested individuals in the control stock. Following the method described above, the flies were selected and bred for five generations.

For the select and control lines, the responsiveness of male progeny in each generation to ME was tested in the laboratory. Firstly, 200 male flies (15 days old) of the select and control lines were randomly chosen as the test subjects. Then the double-exposure experiment method described above was performed to detect the non-responsiveness ratio to ME. Finally, the non-responsiveness to ME ratio of male flies were calculated using the following equation (1). Each experiment consisted of three replicates and was conducted from 9 a.m. to 12 a.m. During experiments, temperature and relative humidity in the room were maintained at 27 ± 1 °C and 75 ± 1% RH, respectively.

1 $${\rm{N}}{\rm{o}}{\rm{n}}{\rm{ \mbox{-} }}{\rm{r}}{\rm{e}}{\rm{s}}{\rm{p}}{\rm{o}}{\rm{n}}{\rm{s}}{\rm{i}}{\rm{v}}{\rm{e}}{\rm{n}}{\rm{e}}{\rm{s}}{\rm{s}}\,{\rm{t}}{\rm{o}}\,{\rm{M}}{\rm{E}}\,{\rm{r}}{\rm{a}}{\rm{t}}{\rm{e}}\,( \% )=\frac{{\rm{N}}{\rm{o}}.{\rm{o}}{\rm{f}}\,{\rm{m}}{\rm{a}}{\rm{l}}{\rm{e}}\,{\rm{f}}{\rm{l}}{\rm{i}}{\rm{e}}{\rm{s}}\,{\rm{n}}{\rm{o}}{\rm{t}}\,{\rm{t}}{\rm{r}}{\rm{a}}{\rm{p}}{\rm{p}}{\rm{e}}{\rm{d}}\,{\rm{b}}{\rm{y}}\,{\rm{M}}{\rm{E}}\,}{{\rm{N}}{\rm{o}}.{\rm{o}}{\rm{f}}\,{\rm{t}}{\rm{e}}{\rm{s}}{\rm{t}}\,{\rm{m}}{\rm{a}}{\rm{l}}{\rm{e}}\,{\rm{f}}{\rm{i}}{\rm{l}}{\rm{e}}{\rm{s}}}{\rm{\times }}{\rm{100}}$$

### Identification of antennal differentially expressed proteins between the responsive and non-responsive males by iTRAQ

#### Protein extraction, digestion and iTRAQ labeling

The antennae were dissected separately from the responsive and non-responsive male adults (each was 18 days old; approximately 4500 individuals); these came from the sixth generation colony of the oriental fruit fly in the select line. Their antennae were frozen immediately in liquid nitrogen, and stored at −80 °C until extraction. Three independent biological replicates of the frozen antennae were prepared for the iTRAQ analysis. The antennae were dissected in a SDT lysis buffer (containing 4% sodium dodecyl sulfate [SDS] and 150 mM Tris-HCl [pH 8.0]), and transferred to 2 mL tubes with an amount of quartz sand (another 1/4 inch ceramic bead MP 6540-424 for the tissue samples). The lysate was homogenized by an MP homogenizer (24 × 2, 6.0 M/S, 60 s; done twice). The homogenate was sonicated and then boiled for 15 min. After being centrifuged at 14000 g for 40 min, the supernatant was filtered with 0.22 µm filters. The filtrate was quantified with a BCA Protein Assay Kit (Bio-Rad, USA). For a quality check, 20 µg of the total proteins per sample were mixed with 5 × Loading Buffer and boiled for 5 min. The proteins were separated on a 12.5% SDS-PAGE gel. Protein bands were visualized through Coomassie Blue R-250 staining.

The extracted proteins were digested according to the previously reported method^35,58,59^. Briefly, 200 μg of proteins for each sample were digested with 4 μg Trypsin Gold (Promega, Madison, WI, USA) in 40 μL DS buffer overnight at 37 °C, and the resulting peptides were collected as a filtrate. The peptides of each sample were desalted on C18 Cartridges (Empore™ SPE Cartridges C18, bed I.D. 7 mm, volume 3 mL, Sigma), concentrated by vacuum centrifugation and reconstituted in 40 µl of 0.1% (v/v) formic acid. The peptide content was estimated by absorbance at 280 nm using an extinction coefficient of 1.1 of 0.1% (g/l) solution that was calculated on the basis of the frequency of tryptophan and tyrosine in vertebrate proteins. After digestion, the peptides were reconstituted in 0.5 M TEAB and labeled according to the manufacturer’s instructions for the iTRAQ 8-plex reagents (Applied Biosystems, Foster City, CA, USA).

#### Liquid chromatography (LC) separation and mass spectra (MS) quantification of peptides

iTRAQ labeled peptides were fractionated by SCX chromatography (Phenomenex, Inc, USA) using a Shimadzu LC-20AB HPLC Pump system. The peptides were eluted with a gradient of buffer A (10 mM NaH_2_PO_4_ in 25% ACN, pH 3.0) and buffer B (500 mM KCl, 10 mM KH2PO4 in 25% of ACN, pH 3.0). The specific fractionating procedures were as follows: a flow rate of 1 ml/min with a gradient of 0–8% buffer B for 22 min, 8–52% buffer B for 30 min, 52–100% buffer B for 50 min, 100% buffer B for 55 min, and buffer B was reset to 0% after 58 min. The elution was monitored by absorbance at 214 nm, and fractions were collected every 1 min. Collected fractions were desalted on C18 Cartridges and concentrated by vacuum centrifugation. Nano LC-MS/MS analysis on each of these fractions was performed using a Q-Exactive mass spectrometer (Thermo Fisher Scientific Inc. Rockford, IL., USA) equipped with nanoelectrospray ionization.

Peptides were identified by searching against the Uniprot_*Bactrocera _dorsalis*_19817_20160321.fasta (19817 sequences, download Mar 21, 2016) using a MS/MS data interpretation algorithm within MASCOT search engine (Matrix Science, London, UK; v.2.2) embedded into Proteome Discoverer 1.4 (Thermo, Pittsburgh, USA). When the MASCOT software was used to search the database, 4622 proteins were identified with a false discovery rate (FDR) of less than 1%. Differential expression ratios for proteins were obtained from MASCOT software (http://www.matrixscience.com), which calculates protein ratios using only ratios from the spectra that are distinct for each protein and excluding the shared peptides of protein isoforms. To calculate the differential expression ratios, all identified spectra from a protein were used to obtain an average protein ratio that was relative to the control label. Student *t*-test was used to analyze the differentially expressed proteins in antennae between the responsive and non-responsive *B. dorsalis* male flies. We only identified proteins with *P*-values <0.05 and fold changes ≥1.20 or ≤0.83 as being differentially expressed^60^.

#### Proteins functional and pathway enrichment analyses

Functional annotation of the proteins identified in *B. dorsalis* was carried out using Blast2GO, an integrated GO annotation and data mining tool that assigns GO through BLAST searches against online protein databases (http://www.geneontology.org)^61,62^. GO enrichment analysis was performed to provide all the GO terms that were significantly enriched in the differentially expressed proteins. Specifically, gene ontology enrichment analyses were performed using a hypergeometric test to map all differentially expressed proteins to GO terms in the database (http://www.geneontology.org/). The test uses the following equation (2):

2 $$P=1-\sum _{i=0}^{{\rm{m}}-1}\frac{(\begin{array}{c}M\\ i\end{array})(\begin{array}{c}N-M\\ n-i\end{array})}{(\begin{array}{c}N\\ n\end{array})}$$

where *N* is the number of all proteins with GO annotation; *n* is the number of differentially expressed proteins in *N*; *M* is the number of all proteins annotated to certain GO terms; and *m* is the number of differentially expressed proteins in *M*. The calculated *P*-value was first subjected to a Bonferroni correction, taking a corrected *P*-value of 0.05 as a threshold for statistical significance. GO terms fulfilling this condition were defined as significantly enriched GO terms in differentially expressed proteins. All identified proteins were mapped to pathway in the Kyoto Encyclopedia of Genes and Genomes (KEGG) database using KEGG Automatic Annotation Server software (http://www.genome.ad.jp/kegg/). Significantly enriched metabolic pathways or signal transduction pathways in differentially expression proteins were identified using the same calculating formula as in GO analysis. Here *N* represents the number of all proteins with KEGG annotation, *n* is the number of differentially expressed proteins in *N*, *M* is the number of all proteins annotated to specific pathways, and *m* is number of differentially expressed proteins in *M*.

### Relative expression analysis of the candidate olfactory proteins at transcript levels by quantitative real-time PCR

The expression of protein transcripts was studied by qRT-PCR to validate the proteomic results. Specific olfaction genes that encode the olfactory proteins were detected following methods described previously^2,63^. An RNA extraction kit (Takara Biotechnology Co., Ltd., Japan) was used to extract the total RNA from the non-responsive and responsive male files’ antennae following the manufacturer’s instructions, and a gDNA eliminator spin column removed the genomic DNA. RNA was quantified by measuring the absorbance at 260 nm in a spectrophotometer (Thermo Nano Drop^TM^ 2000c; Santa Clara, USA). The purity of all RNA samples was assessed at an absorbance ratio of OD_260/280_, while the integrity of RNA was verified by 1% agarose gel electrophoresis. The cDNA was synthesized by using a PrimeScript^TM^ RT reagent Kit (Takara Biotechnology Co., Ltd., Japan), subsequently serving as a template for qRT-PCR.

The gene-specific primers were designed in Primer 5.0 (Premier, Canada) (Table S2). These were used to conduct quantitative real-time PCR to detect the differential expression olfaction proteins of responders compared with non-responders at the transcription level. By using a SYBR Premix ExTaq kit (Tiangen, Guangzhou, China) following the manufacturer’s instructions, with a Stratagene Mx3000 P thermal cycler (Agilent Technologies, Wilmington, Germany). The PCR efficiency of the genes was validated before gene expression analysis. The PCR master mix (20 μL) contained 10 μL of SYBR Green Supermix, 1 μL of cDNA templates, 1 μL of each forward and reverse primer (1 μmol/L), and 7 μL of double-distilled water. The following thermal program was executed: 95 °C for 15 min, followed by 40 cycles of 95 °C for 10 s, 55 °C for 20 s, 72 °C for 20 s, and a final melting cycle (from 55 °C to 95 °C). Three biological and three technical replicates were used and performed for each experiment, respectively. The *α-tubulin* gene of *B. dorsalis* was used as an internal control (GenBank accession number: XM_011212814). The relative gene expression levels were calculated by using the 2^*−ΔΔCT*^ method as described previously^64^.

### Sequence analysis of *BdorOBP2*

The sequences information of *BdorOBP2* was obtained from the National Center for Biotechnology Information (NCBI) database and submitted by Zheng *et al*.^1^ (GenBank accession number: KC559113). The amino acid sequences of *BdorOBP2* identified in this study, along with the 91 known OBPs from other Tephritidae insects, were used to construct a phylogenetic tree with the MEGA 7.0 software (Molecular Evolutionary Genetics Analysis, Version 4.0, Sudhir Kumar, USA) and Interactive Tree Of Life (iTOL) web tool (http://itol.embl.de). The highly divergent signal peptides in the N terminal were removed. Branch support was assessed by the Neighbor Joining technique with 1000 bootstrap replications.

### Responsiveness of immature and mature flies to ME, and *BdorOBP2* expression analysis by quantitative real-time PCR

Each sample of 200 3-day- and 15-day-old males, as well as the 15-day-old females of the control line, were randomly selected as the test subjects and released into a screen cage (1.0 m × 1.0 m × 1.0 m) lacking a trap. Approximately 30 min later, a fly trap containing 1.0 mL of pure ME was placed inside the screen cage. For the controls, traps were also placed but without ME. After trapping for 2 h, we removed the traps and counted the number of attracted flies. Three biological replicates were performed. Additionally, from the untested individuals in the control line, the total RNA of antennae from the 3-day- and 15-day-old males, and the 15-day-old females were extracted to determine their expression of *BdorOBP2* (by using qRT-PCR, as described above).

### Effect of ME regulation on *BdorOBP2* expression in male antennae

The protocol followed published procedures^14^. Each sample of 200 mature males of the control line were randomly selected and placed in a cage (35 cm × 35 cm × 35 cm) without a trap. After being starved for 12 h, 500 μL of ME (1:1 dilution with mineral oil), was applied on a filter paper spotted in a petri dish (diam. = 3.5 cm) to feed the hungry male flies, while the control groups were treated with an equal volume of just the mineral oil. Total RNA was extracted from the antennae after treatment for 1 h and 2 h, then reverse transcribed into single-chain cDNAs. Next, the qRT-PCR quantitative technique was applied to analyze the expression of *BdorOBP2* after the ME treatment. Each treatment was replicated three times.

### RNA interference knock-down of *BdorOBP2*

An RNA interference experiment was performed to demonstrate the role of *BdorOBP2* in ME perception. Double-stranded RNA (dsRNA) was synthesized and purified according to the MEGAscript^®^ RNAi Kit (Thermo Fisher Scientific, USA). Primers used to synthesize dsRNA are listed in Table S3. The GFP gene was used as a control dsRNA (ds*GFP*) (GenBank accession number: AHE38523). The dsRNA expression and injection procedure for the male flies were performed as reported in previous studies^65–67^. Both ds*BdorOBP2* and ds*GFP* were then purified and re-suspended in 1 × *Injection Buffer* (10 mM Tris-HCl pH 7, 1 mM EDTA). Finally, the quality of the dsRNA was determined by 2% TBE gel, and its concentration determined by a Nanodrop 1000 (Thermo, USA).

Each sample of 50 mature males (15 days-old) were randomly selected and placed into a cage (35 cm × 35 cm × 35 cm). The treatment males were then injected with 400 nL of *dsBdorOBP2* (2000 ng/μL). Negative control groups were injected with an equal volume of ds*GFP* and 1 × *Injection Buffer*. The male flies were normally fed to serve as the blank control groups. All flies were reared in the cage and supplied with identical food and water. After treatment for 24 h and 48 h, the respective numbers of dead adults were counted. For the bioassay, males from the ds*BdorOBP2* treatment groups, the negative control groups and the blank control groups were placed inside a screen cage (1.0 m × 1.0 m × 1.0 m) equipped with ME as a trap. The lured males were counted after 2 h, and the silencing efficiency of *BdorOBP2* was detected by qRT-PCR. Each experiment was replicated five times, and 50 flies were tested in each repeat made.

### Statistical analysis

All the results from experimental replicates were expressed as the mean ± SE and analyzed by SAS 9.20 software (SAS Institute Inc. Cary. NC). All data were analyzed using Shapiro-Wilk and Levene’s tests for normal distribution and homogeneity of variances, respectively. If data were normally distributed and had similar variances, then means of measured variables were compared by one-way analysis of variance (ANOVA). Significant ANOVA results multiple comparison over three groups were assessed by Duncan’s multiple range test (DMRT, *P* = 0.05), and two-sample analysis was performed using Student’s *t*-test (*P* = 0.05). Non-normally distributed data were analyzed using a nonparametric Kruskal-Wallis test to compare medians; differences significant at the 0.05 significance level were subjected to a Mann-Whitney test for pairwise comparisons. *P* < 0.05 was considered to be statistically significant. Results were plotted with *Origin* 9.0.

## Electronic supplementary material

Supplementary Information

Dataset 1
