# Supplementary material for: BdorOBP2 plays an indispensable role in the perception of methyl eugenol by mature males of Bactrocera dorsalis (Hendel)
Source: Sci Rep. 2017 Nov 21;7:15894. doi: 10.1038/s41598-017-15893-6 (PMC5698463; doi:10.1038/s41598-017-15893-6)
Supplement: Supplementary file 1 — Supplementary Information [file 41598_2017_15893_MOESM1_ESM.pdf]

BdorOBP2 plays an indispensable role in the perception of methyl eugenol by mature males of *Bactrocera dorsalis* (Hendel)

Huan Liu[1], Xiao-Feng Zhao[1], Lang Fu[2], Yi-Ye Han[1], Jin Chen[1], Yong-Yue Lu[1]*

Odorant binding protein 2

(Protein Accession Number: S5R7H8; Peptides Sequence: gTDEcDTAFQIR)

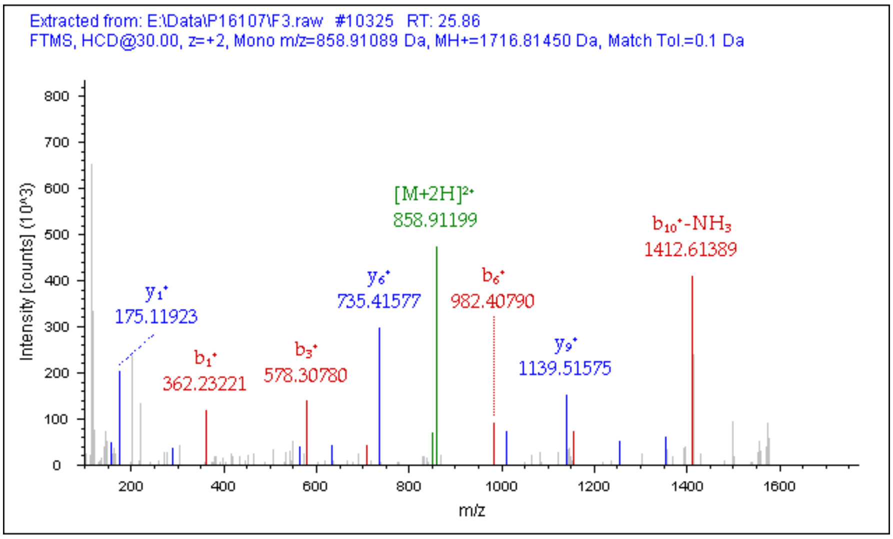

Odorant binding protein 69a

(Protein Accession Number: A0A0G2UEV0; Peptides Sequence: aTGELPNNQNLk)

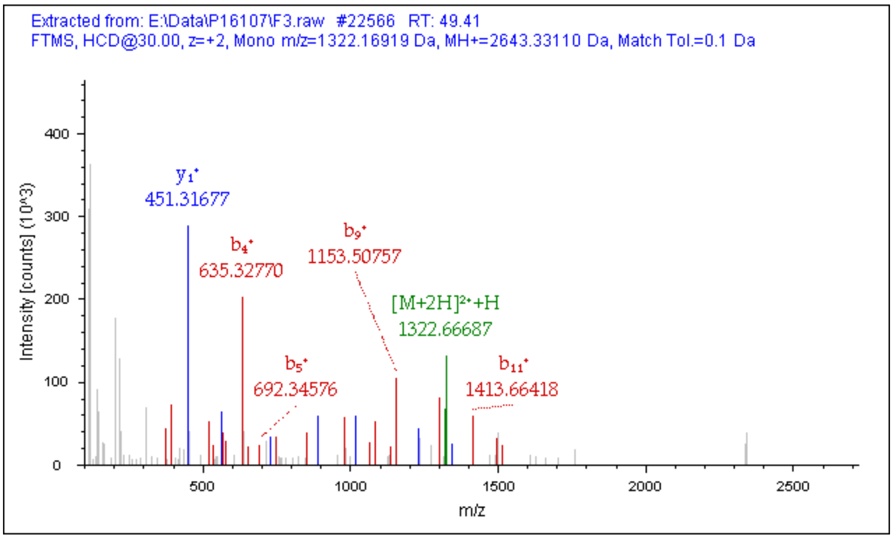

Odorant receptor 94b

(Protein Accession Number: A0A0G2UEY4; Peptides Sequence: tASANIIIAVLk)

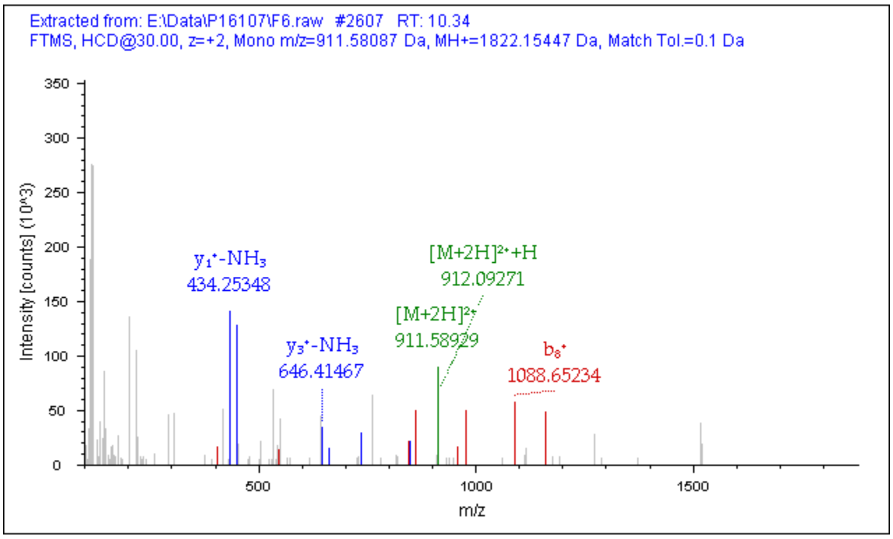

Odorant binding protein 44a

(Protein Accession Number: A0A0G3Z7T5; Peptides Sequence: tQNLIAQLGQNk)

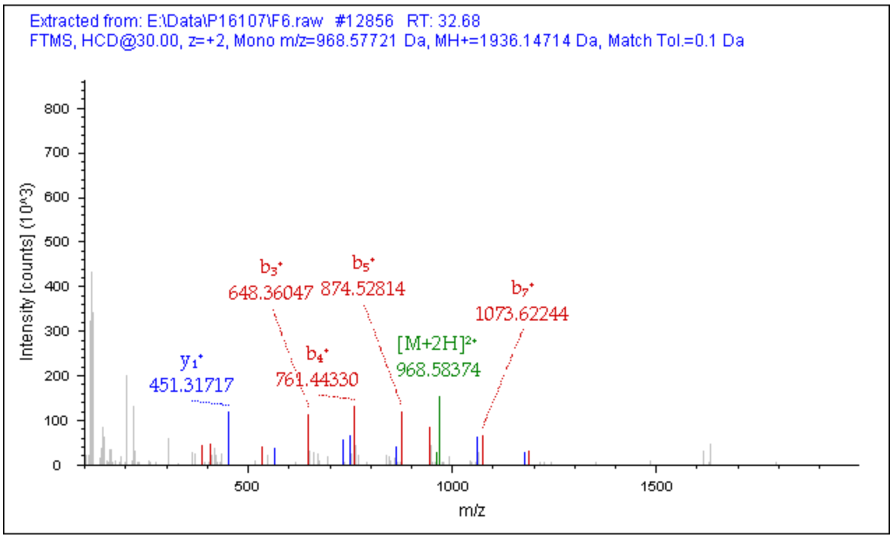

Putative odorant-binding protein A5

(Protein Accession Number: A0A034WGF4; Peptides Sequence: kYDMELVAGNIFTSR)

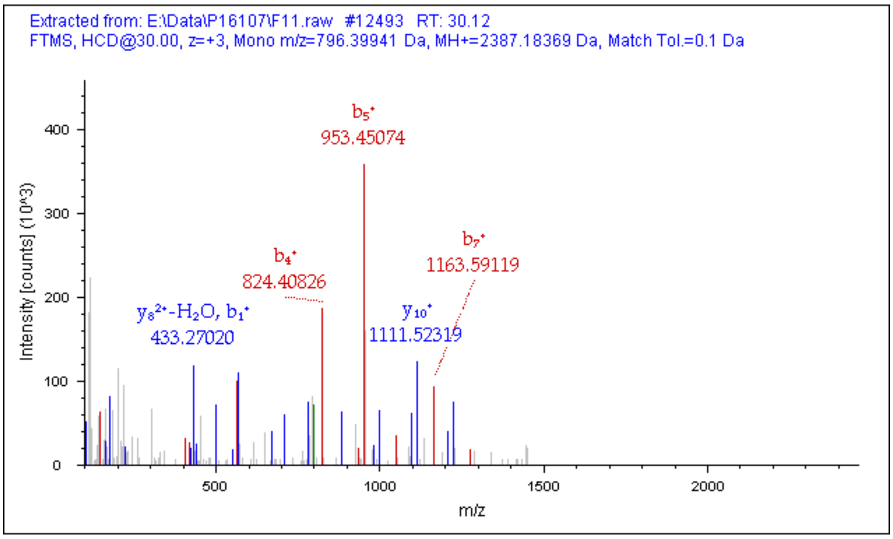

Figure S1. Representative MS spectrum of eight peptides

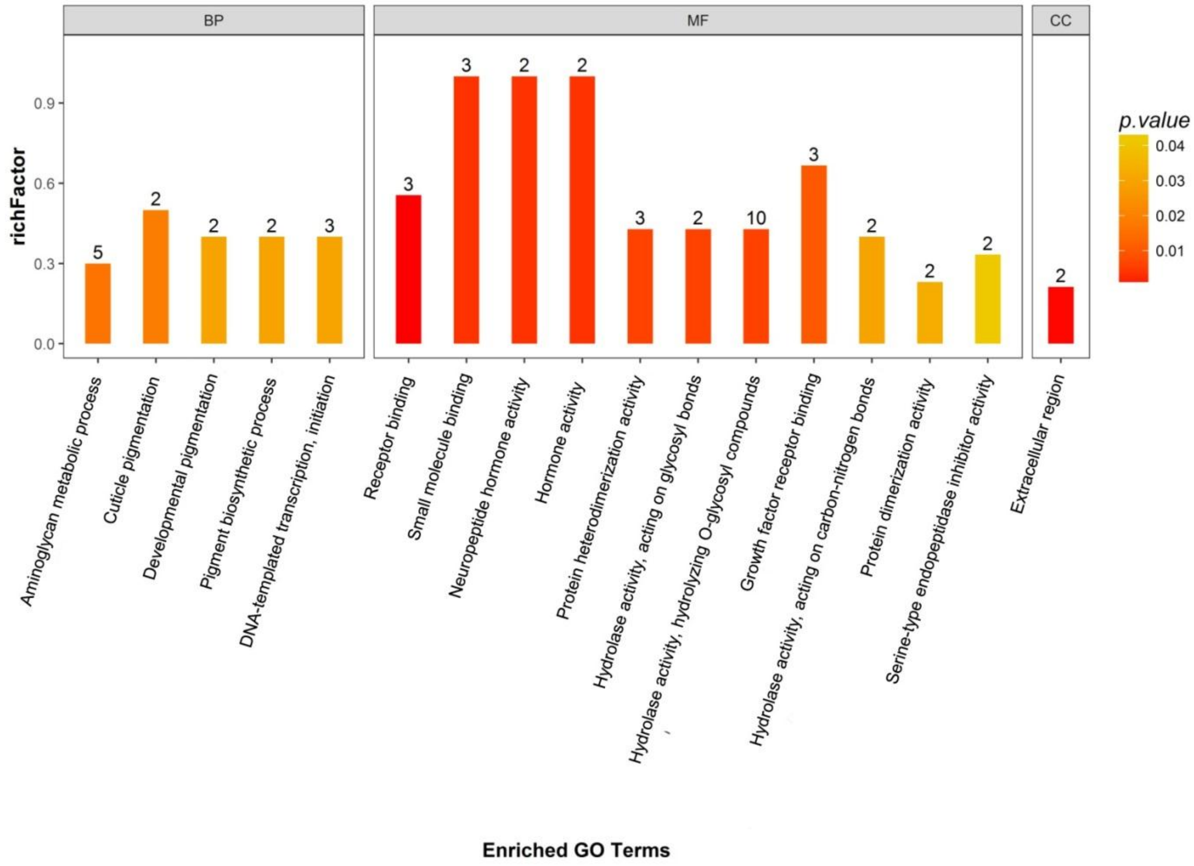

Figure S2. GO enrichment analysis of differentially expressed proteins between the responsiveness and non-responsiveness male flies' antenna. The *x*-axis represents the name of the GO terms, and *y*-axis represents the rich factor. The numbers on the columns represent the protein number. The color of column represents the corresponding *p*-value, and a corrected *p*-value < 0.05 were considered the most significant functional subcategories.

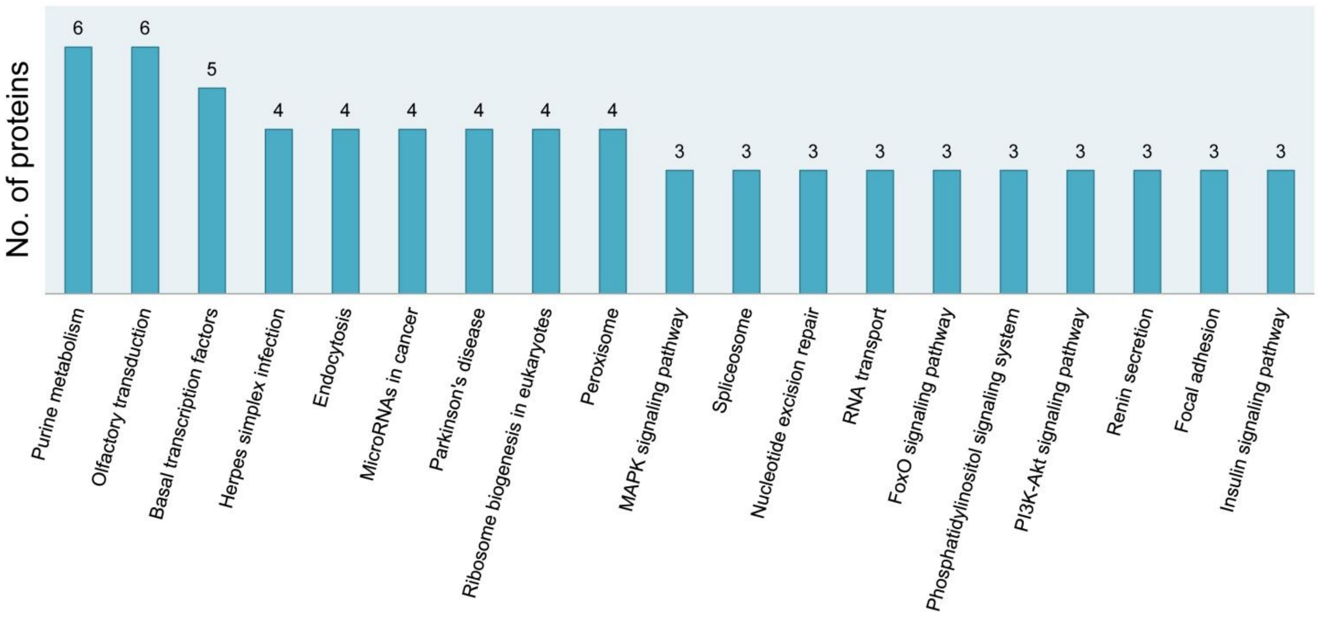

Figure S3. The most represented KEGG pathways in *B.dorsalis* proteome. The *x*-axis represents the name of the pathways, and the *y*-axis represents the protein number.

Table S1. Summary of the proteomics sequencing results.

Group Name	Number
Total spectra	266460
Spectra	48059
Peptides	26679
Unique peptides	25550
Protein	4622
Differentially expressed protein	277

Table S2. Primers used for gene expression detection by qPCR.

Primer name	Nucleotide sequences (Forward)	Nucleotide sequences (Reverse)	Primer efficiency（%）
BdorOBP44a	ACGCGCTGCTACATTGAGTG	GTGCATGAGTCCGATTTCTG	97.83
BdorOBA5	GGCTAAAGGTTACGTGCTAG	GCCACCAGCTCCATATCGT	95.45
BdorOBP69a	AGTGACCACTCTGGAGGTG	TCCAAGTGCACGATGTTGTC	92.53
BdorOBP2	GTTTTGCTAGCCTTTGTCGC	CTTGCATGCACTTGGAGAAG	95.11
BdorOB19A	TCGGATCAAGTTTCTGGCGG	CCTTTCTTCATCGTTTGCATC	93.43
BdorOB99A	CAATGAAGTGCACAGCGTTC	GTCCGGATAATCAAAGTTGTC	97.24
BdorOBP83a-1	CACGTACAGGCACAGGAAC	GTCACCGTTATCGTCCACC	95.99
BdorOBP15	CTAGTGGATAAGTACAAGGCG	GCTTCCTTTGCGCAATTCTC	92.82
BdorPBP4	CAGTCTTGCTCTCTCACTGG	AACGGTATTGTGTAACTGGC	94.69
BdorPBP2	CTTGATTGTTTGTGTGGCGC	CATCACCATTCATCGCACCG	96.92
BdorOR94b	ACCGTTGTCGTGTTCGCTTG	GCTCAGACGCCAACCAATC	92.89
BdorIR84a	CGATCTGCGCATTGGTATTG	GCCGGTAGAAAATGCTGCAG	94.88
α-tubulin	CGCATTCATGGTTGATAACG	GGGCACCAAGTTAGTCTGGA	94.73

Table S3. Primers used for RNA interference analysis.

Primer name	Nucleotide sequences
dsOBP2-F	TAATACGACTCACTATAGGGAGACCACCTTCTCCAAGTGCATGCAAG
dsOBP2-R	TAATACGACTCACTATAGGGAGACCACAAATCCAAGCCCTCGTGCC
dsGFP-F	TAATACGACTCACTATAGGGGAGACCACACGGCCACAAGTTCAGCGT
dsGFP-R	TAATACGACTCACTATAGGGGAGACCACGACCACTACCAGCAGAACA

Note: The underlined nucleotides sequence is T7 promoter.

Table S4. Identification of ORs in non-responsiveness and responsiveness *B.dorsalis* male antennae.

**Accession Number**	**Protein Description**	**Theor. pI/MW (kDa)**	**Non-responsiveness/ Responsiveness**	**Student's *t*-test *P* value**
A0A0G2UKH1	Odorant receptor 13a	8.309/50.726	1.059	0.0688
A0A034WM57	Odorant receptor 83b	8.499/53.078	1.032	0.277
A0A0G2UGJ8	Odorant receptor 43a-1	6.138/43.552	1.056	0.472
A0A0G2UEX9	Odorant receptor 67c	5.402/46.729	0.954	0.599
A0A0G2UEY4	Odorant receptor 94b	8.558/46.003	1.683	0.0198
